# Trends in the prevalence of elevated cardiovascular risk and the control of its risk factors Among US adults, 2001–2020

**DOI:** 10.3389/fcvm.2023.1153926

**Published:** 2023-06-30

**Authors:** Haitao Huang, Jianhong Liu, Xiao Liang, Lingyan Fang, Chenhui Yang, Kangling Ke, Hemanyun Bai, Weize Xu, Weiyan Li, Fanji Meng, Can Chen

**Affiliations:** ^1^ Department of Cardiology, The Second Affiliated Hospital of Guangdong Medical University, Zhanjiang, China; ^2^ The First Clinical College, Guangdong Medical University, Zhanjiang, China; ^3^ Key Laboratory of Environment and Health, Ministries of Education and Environmental Protection, And State Key Laboratory of Environmental Health (Incubation), School of Public Health, Tongji Medical College, Huazhong University of Science and Technology, Wuhan, Hubei, China

**Keywords:** cardiovascular risk, primary prevention, risk factor profiles, secular trends, national health and nutrition examination survey (NHANES)

## Abstract

**Background:**

An accurate assessment of current trends in cardiovascular risks could inform public health policy. This study aims to determine 20-year trends in the prevalence of elevated cardiovascular risk and its risk factors' control among US adults.

**Methods:**

In this serial cross-sectional analysis of 23,594 adults, aged 40–79 years, without clinical atherosclerotic cardiovascular disease (ASCVD) in the National Health and Nutrition Examination Survey from 2001 to 2020, we calculated the prevalence of elevated cardiovascular risk (10-year ASCVD risk ≥ 7.5%) for all participants and subgroups with their risk factors controlled for diabetes, hypertension, or dyslipidemia.

**Results:**

The age- and sex-adjusted prevalence of elevated cardiovascular risk slightly decreased from 41.5% (95% CI, 39.7–43.3%) in 2001–2004 to 38.6% (95% CI, 36.1–41.1%) in 2017–2020 (*P* for trend = 0.169) while the respective sex-adjusted prevalence significantly increased from 34.4% (95% CI, 32.8–36.0%) to 39.5% (95% CI, 37.0–42.0%; *P* for trend <0.001). Sex and race continued to show disparities in cardiovascular risk. Furthermore, a worsening disparity in age- and sex-adjusted prevalence of elevated cardiovascular risk between young and old and a narrowing gap among different education and poverty index levels (all *P* trend for interaction <0.05). Differential decomposition analysis found that demographic changes (primarily population aging) led to an 8.8% increase in the prevalence of elevated cardiovascular risk from 2001 to 2004 to 2017–2020, while risk factor control led to a 3.8% decrease. The rate of individuals receiving treatment for diabetes, hypertension, or dyslipidemia increased significantly between 2001 and 2020 (all *P* for trend <0.05). The rate of participants with hypertension who achieved blood pressure under 130/80 mmHg and those with dyslipidemia who achieved a non-high-density lipoprotein cholesterol level under 130 mg/dl increased significantly (all *P* for trend <0.001).

**Conclusions:**

There is a slight reduction in the prevalence of age- and sex-adjusted elevated cardiovascular risk among US adults without clinical ASCVD between 2001 and 2020, while the sex-adjusted prevalence significantly increased. The decrease in elevated cardiovascular risk prevalence was mainly attributed to risk factor control, while demographic changes contributed to an increase.

## 1. Introduction

Although cardiovascular outcomes have improved due to advances in medical care and clinical management, cardiovascular disease (CVD) remains the leading cause of morbidity and mortality in the US (1). Furthermore, the total CVD mortality decline rate slowed considerably to under 1% annually in 2011–2018 but increased for age-adjusted stroke mortality in 2016–2017 (2–4). The American Heart Association estimates that 40.5% of the US population will experience CVD by 2030, resulting in total direct medical costs of $818 billion, triple the cost in 2010 (5). CVD prevention is presently a public health priority.

Risk assessment of atherosclerotic cardiovascular disease (ASCVD) is key to CVD prevention and clinical management (6). Estimation using traditional CVD risk factors, including age, sex, race, blood pressure (BP), cholesterol, smoking, and history of diabetes, has been widely used, e.g., in the Pooled Cohort Equations (PCE) (7). PCE is a sex- and race-specific equation that estimates the 10-year ASCVD risk, stratifying the score into four risk groups (<5, 5–7.5, 7.5–20, and ≥20%) (7, 8). Multiple risk stratifications are conducive to good management, but inconvenient for clinical application. A PCE score of 7.5% is a significant threshold for primary prevention. Lipid management guidelines defined adults with PCE score ≥7.5% as “elevated-risk populations” that should consider initiating cost-effective statin therapy for primary prevention (8–12). Understanding the long-term trends in the prevalence of elevated-risk populations and their risk factors' control in ASCVD-free populations by racial and sociodemographic subgroups could help develop evidence-based healthcare policies, programs, and resource allocation.

The purpose of this study was to assess 20-year trends in the prevalence of elevated cardiovascular risk (PCE score ≥ 7.5%) in ASCVD-free US adults and investigate their risk factors' control in individuals with diabetes, hypertension, and dyslipidemia from 2001 to 2020.

## 2. Methods

### 2.1. Data collection

This study utilized data collected from the National Health and Nutrition Examination Survey (NHANES), an ongoing survey that provides health and nutritional information to noninstitutionalized civilians in the US. We included eight 2-year NHANES cycles (2001–2016) and a 4-year pre-pandemic cycle (2017–2020). To minimize the small sample size impact and improve estimates precision, we pooled the survey years into five 4-year periods (13–15). Participants aged 40–79 without clinical ASCVD (any event of coronary heart disease, heart attack, angina, or stroke) were included. Participants with missing data on BP, anti-hypertensive medication use, total cholesterol (TC), high-density lipoprotein cholesterol (HDL-C), smoking status, or a history of diabetes were excluded. The National Center for Health Statistics Institutional Review Board approved the study protocol.

Information about age, sex, race/ethnicity, poverty index, insurance, smoking status, current medication use, and medical conditions was collected during in-home interviews. Mobile examination centers were used to measure weight, height, and BP using standardized techniques. BP was measured three consecutive times using an auscultatory (mercury sphygmomanometer) in 2001–2016 and a digital oscillometer (Omron HEM-907XL, Omron Healthcare) in 2017–2020. We calculated mean systolic and diastolic BP using all available readings. On the basis of the difference between auscultatory and oscillometric devices, we added 1.5 mmHg to oscillometer-measured systolic BP and subtracted 1.3 mmHg from diastolic BP for participants in 2017–2020 to adjust the oscillometer values to those of a mercury sphygmomanometer (14, 16).

TC, HDL-C, triglycerides, and hemoglobin A_1c_ (HbA_1c_) were determined by standard methods using blood samples collected in the mobile examination center. Low-density lipoprotein cholesterol (LDL-C) was calculated using the Friedewald equation (17) when triglycerides were ≤400 mg/dl and the Sampson equation (18, 19) when 400–800 mg/dl. Non–HDL-C was calculated by subtracting HDL-C from TC. Multiplying TC, LDL-C, HDL-C, and non-HDL-C values by 0.0259 to convert them to millimoles per liter. Multiply by 0.0113 to convert triglycerides into millimoles per liter.

### 2.2. Elevated cardiovascular risk

Elevated cardiovascular risk was defined as a 10-year ASCVD risk ≥7.5% using the PCE among ASCVD-free adults aged 40–79 (9, 20, 21). The elevated cardiovascular risk prevalence was assessed by sex, race, and other sociodemographic groups.

### 2.3. Risk factors' control

We included both diagnosed and undiagnosed cases of diabetes, hypertension, and dyslipidemia. Diagnosed cases were identified based on self-reported diagnosis or the use of disease-specific medications. For diabetes, undiagnosed cases were defined as individuals with HbA_1c_ ≥ 6.5%, excluding those who had already received a diagnosis (22). Similarly, undiagnosed hypertension was defined as systolic blood pressure ≥140 mmHg and/or diastolic blood pressure ≥90 mmHg, with diagnosed cases being excluded (23). Dyslipidemia was defined as at least one abnormality in TC, LDL-C, triglycerides, or HDL-C (Supplementary Methods S1) (24, 25).

The prevalence of diagnostic rate (e.g., the proportion of patients diagnosed with diabetes from all those with diabetes), medication use, and clinical risk factors were investigated separately in individuals with diabetes, hypertension, or dyslipidemia. The stringent and relaxed diabetes control targets were HbA_1c_ <7% and <8%, respectively (22, 26). The stringent and relaxed BP control targets were <130/80 and <140/90 mmHg (27). The control targets for dyslipidemia were non-HDL-C < 130 mg/dl and HDL-C ≥ 60 mg/dl. Statins were recommended to individuals with LDL-C > 190 mg/dl or a PCE score ≥ 7.5% with one or more CVD risk factors (e.g., dyslipidemia, diabetes, hypertension, or smoking) (8, 11). TC < 200 mg/dl and LDL-C < 100 mg/dl as lipid control targets for statin recommended populations (25).

### 2.4. Statistical analyses

The unadjusted and adjusted prevalence of elevated cardiovascular risk were calculated separately for each four-year cycle. Age- and sex-adjusted estimates were standardized to the 2017–2018 NHANES data using the direct method and the following eight age and sex groups: ages 40–49, 50–59, 60–69, and 70–79 separately for males and females (Supplementary Methods S2). Considering the skewed distribution of the PCE score, geometric mean was presented for each calendar period. The control of glycemia, BP, and lipids was assessed according to the diagnosis rate, medication use, and achieved rate. We performed subgroup analyses by sex (male/female), race/ethnicity (non-Hispanic White, non-Hispanic Black, Hispanic, and other race), age (40–49, 50–59, 60–69, and 70–79 yeas), education levels (< high school, high school, and > high school), body mass index [<25.0, 25.0–29.9, and ≥30.0 (obesity) kg/m^2^], poverty index (<1.3, 1.3–3.49, and ≥3.50), insurance (uninsured/insured), current smoker (no/yes), and comorbidity (hypertension, diabetes, and dyslipidemia).

Linear trends were assessed using weighted regression, modeling the midpoint of each period. Trends for subgroup differences were assessed using the weighted likelihood ratio test by incorporating an interaction term between calendar year and sociodemographic subgroup in the regression models. Sex-specific logistic regression models assessed the associations between the survey period (a categorical variable) and the prevalence of elevated cardiovascular risk as a function of the survey period, adjusted for age and then further for race/ethnicity, education level, poverty index, and insurance status. Subsequently, the model was run without age to determine the impact of aging on trends in elevated cardiovascular risk prevalence. The contribution of population aging and other influencing factors to the change in the elevated cardiovascular risk prevalence was estimated using differential decomposition (Supplementary Methods S3) (28).

All analyses were conducted using the “survey” package in R software (version 4.1.2). Sample weights, clustering, and stratification were used to obtain nationally representative estimates. Two-sided *P* < 0.05 was considered statistically significant.

## 3. Results

### 3.1. Baseline characteristics of participants

Of this study (*n* = 31,528), we excluded those with clinical ASCVD (*n* = 4,136) or missing data on estimating 10-year ASCVD risk (*n* = 3,798), resulting in a total of 23,594 participants (representing 105.3 million non-institutional residents of the US; Supplementary Figure S1) for analysis. The mean age of enrolled participants was 54.9 (10.2) years, and 12,179 (52.6%) were female (Supplementary Table S4). Missing data on education level [*n* = 17 (0.1%)], body mass index (*n* = 263 [1.1%]), poverty index (*n* = 2,092 [8.9%]), insurance status (*n* = 56 [0.2%]), HbA_1c_ (*n* = 36 [0.2%]), and LDL-C (*n* = 118 [0.5%]). Participants with an education level below high school declined from 15.9% in 2001–2004 to 10.4% in 2017–2020, and those with obesity increased from 33.2% to 43.4% (Table 1). Living in poverty was noted in 14.3–17.3% of the participants, 84.0–90.4% had health insurance, and current smokers decreased from 21.3% to 15.1%.

**Table 1:** Characteristics of participants without clinical ASCVD among US adults, 2001–2020^a^.

	No. of Participants (Weighted %) by Calendar Period^b^					
	2001–2004	2005–2008	2009–2012	2013–2016	2017–2020	*P* for
Characteristics	(*n* = 4,202)	(*n* = 4,772)	(*n* = 5,291)	(*n* = 5,395)	(*n* = 3,934)	trend^c^
Age, mean (SD), y	53.7 (10.3)	54.3 (10.2)	55.0 (10.1)	55.8 (10.2)	56.6 (10.3)	<0.001
Sex						0.227
Male	2,106 (48.7)	2,329 (47.2)	2,561 (47.2)	2,510 (46.9)	1,909 (47.2)	
Female	2,096 (51.3)	2,443 (52.8)	2,730 (52.8)	2,885 (53.1)	2,025 (52.8)	
Age group, y						
40–49	1,433 (42.9)	1,496 (38.4)	1,695 (35.4)	1,696 (32.6)	1,095 (30.0)	<0.001
50–59	989 (29.6)	1,274 (32.5)	1,462 (32.5)	1,527 (32.0)	1,100 (31.0)	0.504
60–69	1,041 (16.8)	1,214 (18.3)	1,386 (21.6)	1,427 (23.7)	1,177 (25.9)	<0.001
70–79	739 (10.7)	788 (10.8)	748 (10.5)	745 (11.7)	562 (13.1)	0.008
Race and ethnicity						
N-H White	2,264 (77.8)	2,381 (75.8)	2,221 (72.6)	1,954 (69.4)	1,330 (67.2)	<0.001
N-H Black	787 (9.1)	1,005 (9.7)	1,167 (9.9)	1,124 (10.3)	1,073 (10.1)	0.501
Hispanic	1,013 (9.1)	1,215 (9.4)	1,395 (11.4)	1,536 (12.6)	870 (13.6)	0.011
Other Race	138 (4.1)	171 (5.0)	508 (6.1)	781 (7.7)	661 (9.1)	<0.001
Education level^d^						
<High school	1,210 (15.9)	1,372 (16.7)	1,451 (17.3)	1,299 (13.8)	698 (10.4)	<0.001
High school	1,012 (26.0)	1,128 (25.5)	1,174 (21.7)	1,161 (20.5)	919 (26.0)	0.06
>High school	1,978 (58.0)	2,268 (57.8)	2,659 (61.0)	2,935 (65.7)	2,313 (63.7)	<0.001
BMI^d^, kg/m^2^						
<25.0	1,074 (28.1)	1,214 (28.5)	1,291 (26.4)	1,358 (24.8)	845 (22.1)	<0.001
25.0–29.9	1,629 (38.7)	1,707 (35.3)	1,867 (36.2)	1,781 (34.1)	1,310 (34.5)	0.002
≥30.0	1,401 (33.2)	1,802 (36.2)	2,082 (37.4)	2,212 (41.0)	1,758 (43.4)	<0.001
Poverty index^d,e^						
<1.30	926 (15.1)	1,104 (14.3)	1,423 (17.1)	1,441 (17.3)	832 (14.4)	0.476
1.30–3.49	1,455 (32.7)	1,596 (31.9)	1,691 (33.0)	1,770 (32.0)	1,285 (31.8)	0.713
≥3.50	1,555 (52.2)	1,742 (53.8)	1,679 (49.9)	1,696 (50.7)	1,307 (53.8)	0.848
Insured	3,548 (88.3)	3,891 (86.4)	4,123 (84.0)	4,501 (87.9)	3,414 (90.4)	0.08
Current smoker	906 (21.3)	1,011 (20.9)	1,015 (18.0)	991 (17.0)	684 (15.1)	<0.001
Dyslipidemia^f^	2,734 (63.8)	3,307 (68.6)	3,632 (67.7)	3,784 (69.1)	2,689 (67.4)	0.033
Hypertension	2,162 (44.9)	2,394 (45.2)	2,626 (44.0)	2,763 (47.4)	2,127 (47.7)	0.066
Diabetes	638 (11.0)	846 (12.3)	1,023 (13.7)	1,148 (16.6)	917 (17.5)	<0.001

ASCVD, atherosclerotic cardiovascular disease; SD, standard deviation; N-H, non-Hispanic; BMI, body mass index.

^a^ ASCVD-free adults aged 40–79 years with all variables of the Pooled Cohort Equations were enrolled.

^b^ Values are numbers (weighted percentages), unless specified as mean (SD).

^c^ The statistical significance of a linear trend from 2001 to 2004 through 2017 to 2020 was assessed using weighted regression and modeling the midpoint of each time period.

^d^ Missing values for education level, BMI, poverty index, and insurance status were 17, 263, 2092, and 56 participants, respectively.

^e^ Represents the ratio of family income to the federal poverty threshold, adjusting for household size. A higher ratio indicates a higher level of income.

^f^ Dyslipidemia was defined as at least one abnormality in TC, LDL-C, TG, or HDL-C or self-reported diagnosis of high cholesterol level or taking lipid-lowering drugs.

### 3.2. Trends in 10-year ASCVD risk

The projected 10-year ASCVD risk score showed a right-skewed distribution, in males significantly larger than in females (Supplementary Figure S2). The age- and sex-adjusted 10-year ASCVD risk geometric mean decreased from 5.1% (95% CI, 4.9%–5.4%) in 2001–2004 to 4.6% (95% CI, 4.4%–4.8%) in 2009–2012, but then rose to 4.7% (95% CI, 4.4%–5.0%) in 2017–2020 (*P* for linear trend = 0.054), with similar trends for males and females. The 10-year ASCVD risk showed a significant decrease in Hispanic during 2001–2020 (*P* for linear trend = 0.027), while it slightly increased in non-Hispanic Black (Supplementary Table S1).

### 3.3. Trends in elevated cardiovascular risk

**Figure 1:**
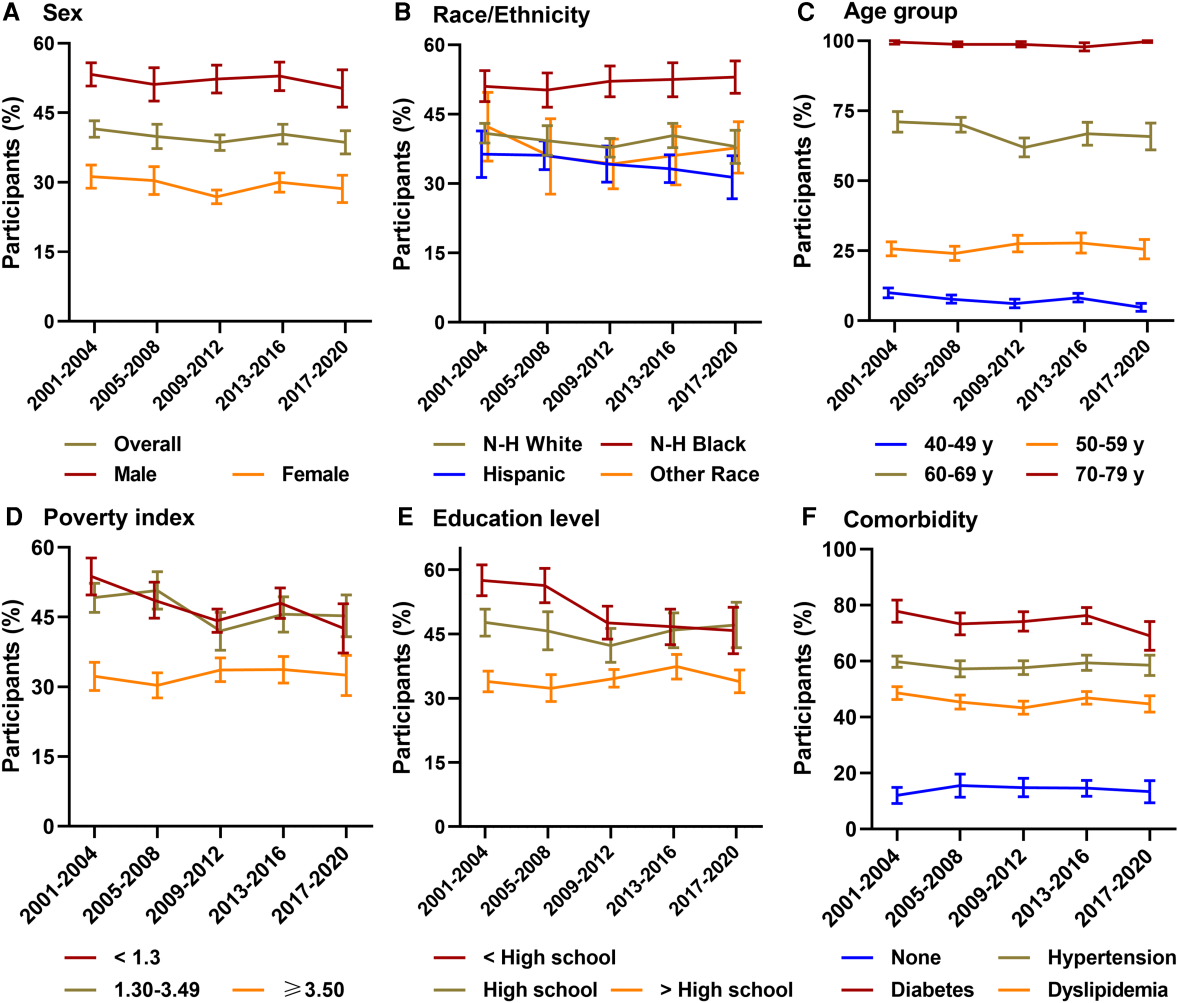
Prevalence of elevated cardiovascular risk among US adults, 2001–2020. N-H, Non-Hispanic; ASCVD, atherosclerotic cardiovascular disease. Survey-weighted national proportion (line) and 95% CIs (error bars) are shown (**A**) overall and by sex, (**B**) race/ethnicity, (**C**) age group, (**D**) poverty index, (**E**) education level, and (**F**) comorbidity. Elevated cardiovascular risk was defined as a projected 10-year ASCVD risk of 7.5% or greater using the Pooled Cohort Equations. All estimates were standardized to the 2017–2018 NHANES ASCVD-free adults by the direct method. For males and females, the estimates were age-adjusted, and for other groups, it was age- and sex-adjusted. In comorbidity, none was defined as participants without dyslipidemia, hypertension, and diabetes.

The age- and sex-adjusted prevalence of elevated cardiovascular risk had slightly decreased from 2001 to 2004 (41.5% [95% CI, 39.7–43.3%]) to 2017–2020 (38.6% [95% CI, 36.1–41.1%]) (*P* for linear trend = 0.169; Figure 1, Supplementary Table S2). Similar trends were seen in males and females. During 2001–2020, there was a significant decline among participants aged 40–49 (*P* for linear trend = 0.001) and 60–69 years (*P* for linear trend = 0.032), a relatively stable trend among those aged 50–59 and 70–79 years. Similar declining trends were noted when analyzed by age, sex, education, body mass index, poverty index, and insurance. However, we noted a worsening disparity between young and old and a narrowing gap among education and poverty index levels (all *P* trend for interaction <0.05; Supplementary Table S2). The elevated cardiovascular risk improved significantly among the less-educated and the poor and slightly among the more-educated and wealthy participants.

The sex-adjusted prevalence of elevated cardiovascular risk significantly rose through 2001–2020 from 34.4% (95% CI, 32.8–36.0%) to 39.5% (95% CI, 37.0–42.0%) (*P* for linear trend <0.001; Supplementary Table S3). It had a 6.4% (95% CI, 1.6–11.3%) absolute increase in males and a 4.0% (95% CI, 0.4%–7.7%) increase in females during 2001–2020. Males had a higher prevalence of elevated cardiovascular risk than females (all *P* for group difference <0.001), females were nearly half as likely to have an elevated cardiovascular risk in 2017–2020 (29.4% [95% CI, 26.3–32.4%]) as males (51.2% [95% CI, 47.2–55.2%]). Non-Hispanic Black were more likely than Hispanic and non-Hispanic White to have an elevated cardiovascular risk (all *P* for group difference <0.05). There is a largest absolute change in prevalence of elevated cardiovascular risk among non-Hispanic Black (10.7% [95% CI, 6.0–15.5%]) and a smallest absolute change among Hispanic (2.4% [95% CI, −4.1%–8.9%]) during 2001–2020.

As shown in Table 2, the prevalence of elevated cardiovascular risk showed a significant negative trend in the age- and sex-adjusted overall model and the age-adjusted sex-specific models (all *P* for linear trend <0.05). The linear trends for overall and males, but not females (*P* for linear trend = 0.06), remained significant when the models were additionally adjusted for race/ethnicity, education level, poverty index, and insurance. The overall and sex-specific models showed a significant positive linear trend when age was excluded to account for ageing impact (all *P* for linear trend < 0.05).

**Table 2:** Weighted associations of calendar period and prevalence of elevated cardiovascular risk by sex^a^.

Calendar Period	Sample size^b^	Adjusted for Age	Multivariable Adjusted^c^	Multivariable Adjusted, except Age
Both sexes^d^				
2001–2004	3,931	1.00 (reference)	1.00 (reference)	1.00 (reference)
2005–2008	4,440	0.82 (0.69–0.98)	0.80 (0.67–0.96)	1.02 (0.89–1.17)
2009–2012	4,789	0.69 (0.57–0.83)	0.65 (0.53–0.80)	1.08 (0.94–1.23)
2013–2016	4,900	0.80 (0.66–0.96)	0.78 (0.65–0.92)	1.33 (1.17–1.51)
2017–2020	3,417	0.64 (0.53–0.78)	0.63 (0.51–0.78)	1.36 (1.16–1.61)
*P* for linear trend^e^		<0.001	<0.001	<0.001
Male				
2001–2004	1,984	1.00 (reference)	1.00 (reference)	1.00 (reference)
2005–2008	2,162	0.75 (0.60–0.95)	0.74 (0.58–0.94)	1.01 (0.85–1.20)
2,009–2012	2,333	0.75 (0.59–0.97)	0.70 (0.54–0.90)	1.22 (1.03–1.45)
2013–2016	2,290	0.76 (0.61–0.96)	0.72 (0.57–0.90)	1.38 (1.18–1.60)
2017–2020	1,655	0.61 (0.48–0.78)	0.57 (0.44–0.73)	1.40 (1.14–1.71)
*P* for linear trend^e^		<0.001	<0.001	<0.001
Female				
2001–2004	1,947	1.00 (reference)	1.00 (reference)	1.00 (reference)
2005–2008	2,278	0.93 (0.73–1.19)	0.94 (0.74–1.20)	1.03 (0.86–1.24)
2009–2012	2,456	0.61 (0.50–0.75)	0.61 (0.49–0.76)	0.91 (0.78–1.06)
2013–2016	2,610	0.85 (0.65–1.12)	0.88 (0.68–1.15)	1.27 (1.07–1.51)
2017–2020	1,762	0.68 (0.52–0.90)	0.73 (0.54–0.99)	1.32 (1.07–1.64)
*P* for linear trend^e^		0.01	0.06	0.001

^a^ Elevated cardiovascular risk was defined as a projected 10-year risk of atherosclerotic cardiovascular disease of 7.5% or greater using the Pooled Cohort Equations.

^b^ Total sample size for male and female are 10,424 and 11,053 after excluded participants with missing values for education level, poverty index, and insurance status.

^c^ Adjusted for age, race/ethnicity, education level, poverty index, and insurance status, including any significant 2-way interactions.

^d^ All of the models for both sexes combined include sex as a covariate.

^e^ The statistical significance of a linear trend from 2001 to 2004 through 2017–2020 was assessed using the weighted regression and modeling the midpoint of each time period.

### 3.4. Trends in glycemic, blood pressure, and lipids control

The rate of participants with diabetes using anti-diabetic medications increased significantly from 64.4% (95% CI, 60.3–68.5%) in 2001–2004 to 75.9% (95% CI, 72.9–78.9%) in 2013–2016, but subsequently declined to 73.7% (95% CI, 70.3–77.1%) in 2017–2020 (*P* for trend < 0.001; Table 3). Similar trends were seen in medication use for hypertension (*P* for linear trend = 0.018). Medication use for dyslipidemia increased from 26.3% (95% CI, 24.0–28.6%) in 2001–2004 to 36.5% (95% CI, 34.3–38.7%) in 2009–2012 and subsequently leveled off (*P* for linear trend < 0.001). There was a significant improvement for statin therapy from 20.0% (95% CI, 18.0–21.9%) to 36.4% (95% CI, 32.2–40.7%) during 2001–2020 (*P* for linear trend < 0.001). The rate of BP and lipids control showed a significant improvement (*P* for linear trend < 0.001), while glycemic control remained stable during 2001–2020 (*P* for linear trend > 0.05).

**Table 3:** Trends in glycemic, blood pressure, and lipids control among US adults^a^.

	Weighted Prevalence (95% Confidence Interval), %^b^						Absolute
	2001–2004	2005–2008	2009–2012	2013–2016	2017–2020	*P* trend^c^	change^d^, %
Diabetes (*n* = 4,559)							
Diagnostic rate	82.2 (78.5–85.9)	79.4 (74.7–84.1)	82.1 (78.6–85.7)	88.1 (85.1–91.0)	84.6 (81.4–87.8)	0.009	2.4 (−2.5 to 7.2)
Medicine for diabetes	64.4 (60.3–68.5)	66.5 (61.5–71.5)	69.1 (66.2–71.9)	75.9 (72.9–78.9)	73.7 (70.3–77.1)	<0.001	9.3 (4.0 to 14.6)
HbA_1c_ < 8%	75.0 (70.8–79.3)	78.5 (74.8–82.3)	76.9 (72.8–81.0)	73.2 (69.7–76.7)	77.7 (72.9–82.5)	0.814	2.6 (−3.8 to 9.0)
HbA_1c_ < 7%	56.7 (52.5–61.0)	55.5 (50.0–60.9)	54.1 (49.1–59.1)	52.3 (48.1–56.5)	57.7 (51.6–63.7)	0.828	0.9 (−6.5 to 8.3)
Hypertension^e^ (*n* = 12,072)							
Diagnostic rate	78.0 (75.4–80.6)	82.3 (80.4–84.3)	83.6 (81.3–85.8)	85.2 (83.3–87.0)	80.4 (77.7–83.2)	0.022	2.4 (−1.4 to 6.3)
Medicine for hypertension	59.9 (56.1–63.6)	63.9 (61.5–66.3)	67.2 (64.0–70.3)	65.7 (62.5–69.0)	64.8 (62.3–67.4)	0.018	5.0 (0.4 to 9.5)
BP < 140/90 mmHg	46.6 (43.4–49.9)	55.2 (52.6–57.9)	59.9 (57.4–62.4)	58.3 (54.7–62.0)	55.1 (52.1–58.1)	<0.001	8.5 (4.0 to 12.9)
BP < 130/80 mmHg	25.7 (23.0–28.4)	32.4 (29.3–35.5)	38.8 (35.7–41.8)	36.2 (33.0–39.4)	36.3 (33.2–39.5)	<0.001	10.6 (6.5 to 14.7)
Dyslipidemia^f^ (*n* = 16,139)							
Diagnostic rate	58.4 (55.4–61.4)	63.4 (61.3–65.6)	65.2 (62.8–67.7)	69.9 (67.7–72.2)	71.0 (67.6–74.4)	<0.001	12.6 (8.1 to 17.1)
Medicine for dyslipidemia	26.3 (24.0–28.6)	34.7 (32.3–37.2)	36.5 (34.3–38.7)	36.6 (34.4–38.9)	36.6 (32.5–40.6)	<0.001	10.3 (5.6 to 14.9)
Non-HDL-C < 130 mg/dl	16.3 (14.3–18.2)	24.4 (22.6–26.3)	27.4 (25.1–29.7)	31.4 (29.3–33.6)	32.9 (28.6–37.2)	<0.001	16.6 (11.9 to 21.4)
HDL-C ≥ 60 mg/dl	22.7 (20.7–24.7)	24.3 (22.6–26.0)	22.7 (20.1–25.3)	26.5 (24.0–28.9)	25.1 (22.3–27.8)	0.049	2.4 (−1.0 to 5.8)
Recommend statin^g^ (*n* = 10,689)							
Prevalence of statin use	20.0 (18.0–21.9)	28.3 (25.9–30.7)	30.8 (28.0–33.6)	36.3 (33.6–39.0)	36.4 (32.2–40.7)	<0.001	16.5 (11.8 to 21.2)
TC < 200 mg/dl	36.1 (34.0–38.2)	42.4 (39.8–45.1)	48.8 (45.3–52.3)	50.1 (47.9–52.3)	55.4 (50.1–60.8)	<0.001	19.4 (13.6 to 25.1)
LDL-C < 100 mg/dl	19.5 (16.9–22.0)	31.3 (28.6–34.1)	34.2 (30.7–37.8)	38.2 (35.6–40.8)	40.3 (35.6–45.0)	<0.001	20.9 (15.5 to 26.2)

BP, blood pressure; non-HDL, non-high density lipoprotein cholesterol; HDL-C, high-density lipoprotein cholesterol; TC, total cholesterol; LDL-C, low-density lipoprotein cholesterol; ASCVD, atherosclerotic cardiovascular disease.

^a^ Participants aged 40–79 years without clinical ASCVD and all variables of the Pooled Cohort Equations were included. Diagnosed diabetes, hypertension, and dyslipidemia were defined as self-reported diagnosis or taking drugs. To convert TC, LDL-C, HDL-C, and non-HDL-C to mmol/l, multiply values by 0.0259. To convert triglycerides to mmol/l, multiply values by 0.0113.

^b^ Estimates were age- and sex-adjusted by the direct method to the 2017–2018 NHANES adults without pre-existing ASCVD.

^c^ The statistical significance of a linear trend was assessed using the weighted regression and modeling the midpoint of each time period.

^d^ Indicates the absolute increase in prevalence of glycemic, blood pressure, and lipid control between 2001 and 2004 and 2017 to 2020. Values are percentages (95% CIs).

^e^ Hypertension was defined as a self-reported diagnosis of hypertension, currently taking antihypertensive drugs, or diagnosed as systolic blood pressure ≥140 mmHg and/or diastolic blood pressure ≥90 mmHg.

^f^ Dyslipidemia was defined as at least one abnormality in TC, LDL-C, TG, or HDL-C or self-reported diagnosis of high cholesterol level or taking lipid-lowering drugs.

^g^ Recommend statin was defined as (1) LDL > 190 mg/dl or (2) one or more CVD risk factors (i.e., dyslipidemia, diabetes, hypertension, or smoking) with projected 10-year ASCVD risk ≥7.5%.

### 3.5. Differential decomposition analysis

The prevalence of elevated cardiovascular risk increased by 5.0% during 2001–2020 using differential decomposition analysis. This increase was association with an 8.8% increase due to demographic changes (primarily population aging; 11.2% in males and 7.1% in females) and an 3.8% decrease due to risk factor control (5.8% in males and 1.9% in females; Table 4). Risk factor control worked best for non-Hispanic White, whereas aging posed the greatest risk. Risk factor control had little effect among non-Hispanic Black. Their elevated cardiovascular risk increased by 0.5%, and the overall prevalence increased by 10.1% during 2001–2020. Population aging had the least impact among Hispanics, whose risk factors were also well controlled.

**Table 4:** Differential decomposition analysis of the prevalence of elevated cardiovascular risk^a^.

	Prevalence of elevated cardiovascular risk		Influence to the prevalence change^c^		
	2001–2,004	2017–2,020	Absolute change^b^	Population aging	Other factors
Overall	34.6%	39.5%	5.0%	8.8%	−3.8%
Sex					
Male	44.9%	50.3%	5.4%	11.2%	−5.8%
Female	24.7%	29.9%	5.2%	7.1%	−1.9%
Race/ethnicity					
N-H White	34.1%	39.2%	5.1%	10.6%	−5.5%
N-H Black	43.8%	53.9%	10.1%	9.6%	0.5%
Hispanic	30.4%	31.9%	1.5%	5.7%	−4.2%
Other Race	34.8%	39.6%	4.8%	7.7%	−2.9%

*N*-H, non-Hispanic.

^a^ Elevated cardiovascular risk was defined as a projected 10-year risk of atherosclerotic cardiovascular disease of 7.5% or greater using the Pooled Cohort Equations.

^b^ Indicates the absolute decrease in the prevalence of elevated cardiovascular risk between 2,001 to 2004 and 2017 to 2020.

^c^ The difference decomposing was used to estimate the contribution of population aging and other influencing factors to the change in the prevalence of elevated cardiovascular risk.

## 4. Discussion

This cross-sectional study examined 20-year (2001–2020) trends in the prevalence of elevated cardiovascular risk and the control of related risk factors in ASCVD-free participants. The age- and sex-adjusted prevalence of elevated cardiovascular risk slightly decreased, while the sex-adjusted and sex-specific prevalence increased significantly. Sex and race continued to show disparities in elevated cardiovascular risk. Males were more likely to have elevated cardiovascular risk than females, and non-Hispanic Black than non-Hispanic White and Hispanic. Furthermore, we observed socioeconomic differences in elevated cardiovascular risk. Low educational attainment and family income levels were associated with a high prevalence of elevated cardiovascular risk, but this relationship has decreased over time. The prevalence among those with diabetes or advanced age was higher than those with hypertension or dyslipidemia.

Based on these considerations, patients with diabetes, hypertension, and dyslipidemia have higher lifetime risks than those without these conditions; estimating the risk of future ASCVD events and assessing the control of these diseases are crucial (10). Over the past 20 years, BP and lipid control had improved significantly, and the prevalence of hypertension and dyslipidemia slight increased. By contrast, the proportion of participants achieving glycemic control remained stable, and the prevalence of diabetes increased rapidly. Yet, we should take a correct perspective on the progress made in diabetes prevention and management. Diabetes has a higher diagnosis rate, medication use, and control rate than hypertension or dyslipidemia. Dyslipidemia was the most prevalent, while its diagnosis rate, medication use, and control were the lowest (when BP < 130/80 mmHg as a target, the hypertension control rate was the lowest). Considerable progress in tobacco control was noted, and the number of smokers continued to decline. Although risk factor control (i.e., smoking, hypertension, and dyslipidemia) achieved some results, population aging and increased diabetes significantly increased the prevalence of elevated cardiovascular risk.

Secular trends in 10-year ASCVD risk were explored in several studies. The age- and sex-adjusted 10-year ASCVD risk decreased from 7.6% in 1999–2000 to 6.5% in 2011–2012 among US adults aged ≥20 (29). It is worth noting that the PCE is only applicable to participants aged 40–79 when estimating the 10-year ASCVD risk. It can be used to assess lifetime risk beginning at age 21, but it is based on an inadequate database (10). There was a decline in the age-adjusted mean 10-year ASCVD risk from 13.5% in 1999–2000 to 12.0% in 2017–2018 among participants aged 40–79 (30). Differences in the population's age in the two studies led to wide variations in outcomes. Age is the most critical driving factor for CVD, with 10-year ASCVD risk increasing exponentially with age, resulting in a skewed distribution of the risk score (31). Therefore, describing the 10-year ASCVD risk by the arithmetic mean is inappropriate and could result in large deviations. Recently, Jacobs et al. (32) reported that among adults aged 40–75 in the US from 2013 to March 2020, 58.3% had a 10-year ASCVD risk ranging from 7.5% to 20%, while 10.7% had a 10-year ASCVD risk equal to or greater than 20%. Overall, 69.0% of adults are in a state of elevated cardiovascular risk, but they did not provide a detailed description of their trend changes. In the present study, we employed the accepted cut-off value to depict the patterns in future cardiovascular risk, aiming to inform clinical practice, evaluate the prospective burden on communities, and offer more comprehensible evidence for policy development. Considering the variations in cardiovascular risk across diverse populations, it is advisable for policymakers and guideline developers to pay greater attention to males, non-Hispanic Black individuals, as well as individuals from low-income and low-education backgrounds.

Effectively controlling multiple risk factors can decrease the risk of CVD events by 50% or more. However, only a small percentage, less than 20% of patients, are able to achieve the desired targets for risk factor reduction, including plasma lipid levels, BP, glycaemic control, body weight, and non-smoking status (33). In 2019, the leading risk factors for mortality were high systolic blood pressure and smoking, which caused 10.8 million global deaths, accounting for 19.2% of all deaths, and resulted in 8.7 million global deaths, representing 15.4% of all deaths (34). The age-adjusted prevalence of hypertension was 31.5% in 2009–2012 and slightly increased to 32.9% in 2017–2020 among US adults aged above 18 (14). While the prevalence of adults with controlled BP, defined as systolic BP <140 mm Hg and diastolic BP <90 mm Hg, experienced a significant decline, dropping from 52.8% to 48.2% between 2009 and 2020. Muntner et al. (35) found that the age-adjusted estimated proportion with controlled BP decreased from 53.0% in 2009–2010 to 43.7% in 2017–2018. Smoking kills, it is not original but worth repeating. Tobacco smoking was responsible for approximately 14% of all deaths in 2019 (36). In our study, the percentage of current smokers decreased from 21.3% to 15.1% between 2001 and 2020. Despite experiencing a decline of over 1% per year in age-standardized tobacco smoking exposure between 2010 and 2019, tobacco still ranks as the third primary risk factor for attributable disability-adjusted life-years (37).

Diabetes is the leading risk factor for cardiovascular events, resulting in over 100,000 deaths in the US in 2020 (38). From 1988 to 1994 to 2017–2020, the prevalence of total diabetes increased from 6.8% to 14.2% (39). Among US adults, the age-adjusted prevalence of diabetes saw a notable rise, increasing from 9.8% in 1999–2000 to 14.3% in 2017–2018 (40). While there was an improvement in glycemic control (achieving a glycated hemoglobin level of <7%) during the same period, with the percentage of participants reaching this target increasing from 36.7% to 50.4% (13). It should be pointed out that current research primarily focuses on the entire adults, including primary and secondary prevention, whereas our study targets individuals without clinical ASCVD. The present analysis thoroughly investigated several significant cardiovascular risk factors by utilizing the latest available national survey data. Dyslipidemia is a modifiable risk factor for CVD that can be reversed through lifestyle modifications and statins. Among US adults, there was a significant reduction in age-adjusted TC levels, dropping from 197 mg/dl in 2007–2008 to 189 mg/dl in 2017–2018 (41). Age-adjusted LDL-C also significantly improved in the overall population from 116 mg/dl in 2007–2008 to 111 mg/dl in 2017–2018. However, among adults receiving statin therapy, the rates of age-adjusted lipid control did not exhibit a significant change, remaining relatively stable at 78.5% to 79.5% throughout this period. Consistent with our findings, there was a significant improvement in lipid levels. It is important to note, however, that more than half of US adults who require statin therapy have LDL-C levels greater than 100 mg/dl, which is worrisome given the overwhelming evidence of cardiovascular benefits below this concentration (10).

Based on the findings of the Global Burden of Disease project, approximately two-thirds of global deaths in 2020 were attributed to chronic non-communicable diseases. It is projected that by 2030, non-communicable diseases will contribute to 77% of all deaths, with this rise primarily influenced by the aging population in Western societies (37). Globally, the aged population (> 65 years) is growing fast; the old will outnumber the young in nearly every country over the next 40–60 years (42). Demographic changes significantly impact ASCVD burdens. Unfortunately, only 6.8% of the US adults had optimal cardiometabolic health in 2017–2018, decreasing from 1999 to 2000 (43). Coronary heart disease incidence was expected to rise by approximately 26% during 2010–2040 and its prevalence by 47% (44). Due to this deterioration, maintaining a healthy elderly population is a major challenge for societies, and much needs to be done in the US. Reprioritization efforts must be made to reduce the widespread suffering and premature deaths caused by CVD, both limiting healthy and sustainable development in every country. Roth et al. (45) ranked the modifiable risk factors attributing to CVD: high systolic BP, dietary risks, high LDL-C, air pollution, a high body mass index, smoking, hyperglycemia, and kidney dysfunction. For 28 chronic diseases, on average, only 18.5% of the population's susceptibility is attributed to genetic factors (46). In other words, the vast majority of the disease burden is controllable or influenced by modifiable factors. According to the results of the present study, risk prevention in the US should focus on controlling diabetes incidence, reducing obesity, and strengthening the control over hypertension and dyslipidemia. Additionally, a healthy diet and lifestyle, and statin therapy are the most effective means of preventing ASCVD (8). It is necessary to assess the 10-year ASCVD risk in individuals aged 40–75, with statin therapy being the first-line treatment when the cardiovascular risk (PCE scores ≥ 7.5%) is elevated. For clinicians, policymakers, and guideline developers, it is important to focus on targeted interventions for risk factor control and addressing the disparities identified. By understanding the evolving trends in cardiovascular risk and their underlying factors, healthcare providers can develop tailored strategies and interventions to effectively prevent and manage ASCVD.

### 4.1. Limitations

Several limitations were present in this study. First, NHANES is a cross-sectional survey; individual longitudinal changes in cardiovascular risk could not be evaluated. Such changes could improve the accuracy of CVD risk prediction and enhance the evidence base for decision-making concerning preventive measures beyond a single risk score assessment (47). Second, self-reported diagnosis and medication use are susceptible to recall bias and inaccuracy. Third, fasting blood samples (i.e., fasting plasma glucose, serum LDL-C, and triglycerides) and 2-hour plasma glucose were not used since only some participants had these data. Fourth, the PCE has not been validated in Hispanic and Asian populations. Additionally, Asian participants were grouped into other races despite the differences within these groups. Finally, due to the COVID-19 pandemic, data collection was suspended in March 2020, resulting in a smaller sample size for 2017–2020.

### 4.2. Future directions

First, further investigation can focus on developing interventions that address age-related cardiovascular risk and can have significant implications for an aging population. Second, delve deeper into the underlying factors contributing to disparities in cardiovascular risk among different demographic groups, such as sex and race, and evaluate interventions targeted at reducing them. Implementing tailored interventions can help address disparities in cardiovascular risk. Finally, our research primarily investigated the control of traditional risk factors. Future research could explore emerging risk factors such as air pollution, dietary patterns, sedentary behavior, sleep quality, or genetic markers, and their impact on cardiovascular health.

## 5. Conclusions

There is a slight reduction in the prevalence of age- and sex-adjusted elevated cardiovascular risk among US adults without clinical ASCVD between 2001 and 2020, while the sex-adjusted prevalence significantly increased. The rate of BP and lipids control showed a significant improvement, while glycemic control remained stable over this period. The decrease in elevated cardiovascular risk prevalence was mainly attributed to risk factor control, while demographic changes contributed to an increase.

## Data Availability

Publicly available datasets were analyzed in this study. This data can be found here: https://www.cdc.gov/nchs/nhanes/index.htm.
