# Supplementary material for: Trends in the prevalence of elevated cardiovascular risk and the control of its risk factors Among US adults, 2001–2020
Source: Front Cardiovasc Med. 2023 Jun 30;10:1153926. doi: 10.3389/fcvm.2023.1153926 (PMC10347386; doi:10.3389/fcvm.2023.1153926)
Supplement: Supplementary file 1 [file Datasheet1.pdf]

Supplementary Material

Trends in the Prevalence of Elevated Cardiovascular Risk and the Control of its Risk Factors Among US Adults, 2001–2020

Haitao Huang, Jianhong Liu, Xiao Liang, Lingyan Fang, Chenhui Yang, Kangling Ke, Hemanyun Bai, Weize Xu, Weiyan Li, Fanji Meng and Can Chen*

*** Correspondence:** Can Chen: chencan-21@163.com

1 Supplementary Methods

Supplementary Methods 1. Definition of diabetes, hypertension, dyslipidemia, and recommend statin

**Diabetes**	
Diagnosed	Self-reported diagnosis of diabetes or currently taking antidiabetic drugs or insulin.
Undiagnosed	Having a HbA_{1c} value ≥ 6.5% among individuals without diagnosed diabetes.
**Hypertension**	
Diagnosed	Self-reported diagnosis of hypertension or currently taking antihypertensive drugs.
Undiagnosed	Having a systolic blood pressure value ≥ 140 mmHg and/or a diastolic blood pressure value ≥ 90 mmHg among individuals without diagnosed hypertension.
**Dyslipidemia**	
Diagnosed	Self-reported diagnosis of high cholesterol level or currently taking antihyperlipidemic drugs.
Undiagnosed	TC ≥ 240 mg/dL, LDL-C ≥ 160 mg/dL, triglycerides ≥ 200 mg/dL, or HDL-C < 40 mg/dL (< 50 mg/dL for female) among individuals without diagnosed dyslipidemia.
**Recommend statin**	
LDL-C > 190 mg/dL or one or more cardiovascular disease risk factors (dyslipidemia, diabetes, hypertension, or smoking) with projected 10-year atherosclerotic cardiovascular disease risk ≥ 7.5%	

TC, total cholesterol; LDL-C, low-density lipoprotein cholesterol; HDL-C, high-density lipoprotein cholesterol. To convert TC, LDL-C, and HDL-C to mmol/L, multiply values by 0.0259. To convert triglycerides to mmol/L, multiply values by 0.0113.

Supplementary Methods 2. Age- and sex-standard proportions of 2017–2018 NHANES adults without clinical ASCVD

Group	2017 NHANES populations	Proportion among adults aged 40-79 years
Male		
40-49 y	18,583,019	14.92%
50-59 y	19,325,556	15.52%
60-69 y	13,359,953	10.73%
70-79 y	6,690,769	5.37%
Female		
40-49 y	18,953,637	15.22%
50-59 y	21,266,900	17.08%
60-69 y	17,251,591	13.85%
70-79 y	9,115,618	7.32%

NHANES, National Health and Nutrition Examination Surveys; ASCVD, atherosclerotic cardiovascular disease.

Supplementary Methods 3. Differential decomposition analysis methods and procedures

The differential decomposition method converts the comparison of the incidence rate of two populations into a comparison of the difference in the incidence rate of two time points in the same population. It is straightforward, residual-free, and quick to apply. The method may provide an answer to the question of "How much of the incidence difference between populations A and B is caused by the age difference, and how much is caused by other factors? "

The main principle is as follows: Assuming we are going to decompose the difference in population A and population B, CDR^B represents the crude incidence of population B, CDR^A represents the crude incidence of population A, *diff* represents the incidence difference between population B and population A, C represents the age composition of the population, and M represents the age-specific incidence, then:

$$diff = CDR^B - CDR^A = \sum C_X^B M_X^B - \sum C_X^A M_X^A$$

Each term in the formula above was divided into two equal terms. Two additional terms were added and deleted, simultaneously, maintaining a constant *diff* value.

$$diff = \left(\frac{\sum C_X^B M_X^B}{2} + \frac{\sum C_X^B M_X^B}{2}\right) - \left(\frac{\sum C_X^A M_X^A}{2} + \frac{\sum C_X^A M_X^A}{2}\right) + \left(\frac{\sum C_X^B M_X^A}{2} - \frac{\sum C_X^B M_X^A}{2}\right) + \left(\frac{\sum C_X^A M_X^B}{2} - \frac{\sum C_X^A M_X^B}{2}\right)$$

Combined the formula above:

$$diff = \sum C_X^B\left(\frac{M_X^B + M_X^A}{2}\right) - \sum C_X^A\left(\frac{M_X^B + M_X^A}{2}\right) + \sum M_X^B\left(\frac{C_X^B + C_X^A}{2}\right) - \sum M_X^A\left(\frac{C_X^B + C_X^A}{2}\right)$$

$$= \sum(C_X^B - C_X^A)\left(\frac{M_X^B + M_X^A}{2}\right) + \sum(M_X^B - M_X^A)\left(\frac{C_X^B + C_X^A}{2}\right)$$

$$= \sum(\text{differences in age structure} * \text{mean age specific incidence of populations A and B})$$

$$+ \sum(\text{differences in age specific incidence} * \text{mean age of populations A and B})$$

Therefore, it follows that: *diff* = effects of the age compositional difference between populations A and B + effects of other factors.

2 Supplementary Tables

Supplementary Table 1. Trends in projected 10-year ASCVD risk among US adults, 2001–2020 [a]

	**Geometric mean (95% Confidence Interval), %** [b]					***P* for**
**Variable**	**2001–2004**	**2005–2008**	**2009–2012**	**2013–2016**	**2017–2020**	**trend** [c]
**Both sexes**						
Overall	5.1 (4.9–5.4)	4.9 (4.6–5.3)	4.6 (4.4–4.8)	4.8 (4.5–5.1)	4.7 (4.4–5.0)	0.054
N-H White	5.0 (4.7–5.3)	4.9 (4.5–5.4)	4.6 (4.4–4.9)	4.9 (4.5–5.3)	4.7 (4.3–5.1)	0.161
N-H Black	6.6 (6.0–7.2)	6.3 (5.8–6.9)	6.3 (5.8–6.9)	6.4 (5.8–7.1)	6.9 (6.3–7.6)	0.968
Hispanic	4.6 (3.9–5.3)	4.3 (3.9–4.7)	3.9 (3.6–4.4)	4.0 (3.6–4.4)	3.7 (3.2–4.1)	0.027
Other Race	5.3 (4.3–6.5)	4.2 (3.5–5.0)	3.8 (3.3–4.5)	4.0 (3.5–4.6)	4.5 (4.0–5.0)	0.981
**Male**						
Overall	7.8 (7.3–8.3)	7.6 (7.2–8.1)	7.3 (6.9–7.7)	7.5 (7.0–8.1)	7.2 (6.6–7.9)	0.267
N-H White	7.6 (7.0–8.1)	7.5 (7.0–8.1)	7.2 (6.7–7.7)	7.5 (6.8–8.1)	7.1 (6.3–8.1)	0.494
N-H Black	10.2 (9.3–11.2)	9.9 (9.2–10.8)	10.6 (9.9–11.3)	10.8 (9.9–11.7)	10.4 (9.6–11.3)	0.399
Hispanic	7.5 (6.7–8.3)	6.7 (6.0–7.5)	6.2 (5.5–7.0)	6.5 (5.9–7.2)	6.0 (5.2–6.8)	0.011
Other Race	8.8 (6.7–11.7)	7.1 (5.6–9.1)	6.3 (5.3–7.6)	6.8 (5.8–8.0)	7.1 (6.1–8.3)	0.845
**Female**						
Overall	3.6 (3.3–3.8)	3.4 (3.1–3.8)	3.1 (2.9–3.4)	3.3 (3.0–3.5)	3.2 (3.0–3.5)	0.064
N-H White	3.5 (3.2–3.8)	3.4 (3.0–3.9)	3.2 (2.9–3.4)	3.4 (3.1–3.7)	3.3 (3.0–3.6)	0.125
N-H Black	4.6 (3.9–5.4)	4.4 (4.0–4.9)	4.3 (3.8–4.8)	4.3 (3.8–5.0)	5.0 (4.3–5.7)	0.547
Hispanic	2.9 (2.4–3.6)	2.8 (2.5–3.2)	2.6 (2.2–2.9)	2.6 (2.2–2.9)	2.3 (2.0–2.7)	0.206
Other Race	3.8 (2.9–5.0)	2.7 (2.1–3.5)	2.6 (2.0–3.2)	2.4 (2.1–2.8)	2.9 (2.5–3.5)	0.73

ASCVD, atherosclerotic cardiovascular disease; N-H, non-Hispanic.
[a] Projected 10-year risk for ASCVD was calculated using the Pooled Cohort Equations.
[b] All estimates were standardized to the 2017–2018 NHANES ASCVD-free adults by the direct method. For both sexes, the estimates were age- and sex-adjusted, and for sex-specific, it was age-adjusted.
[c] The statistical significance of a linear trend from 2001 to 2004 through 2017 to 2020 was assessed using weighted regression and modeling the midpoint of each time period.

Supplementary Table 2. Trends in elevated cardiovascular risk among US Adults, 2001–2020 [a]

Variable	Weighted Prevalence (95% Confidence Interval), % [b]					Prevalence Change [c], %	*P* value	
	2001–2004	2005–2008	2009–2012	2013–2016	2017–2020		Linear [d]	Interaction [e]
**Overall**	41.5 (39.7–43.3)	39.9 (37.3–42.5)	38.6 (36.9–40.2)	40.4 (38.3–42.5)	38.6 (36.1–41.1)	2.9 (-0.2 to 5.9)	0.169	-
**Age group, y**								0.006
40-49	10.0 (8.2–11.7)	7.7 (6.3–9.2)	6.1 (4.6–7.7)	8.2 (6.7–9.8)	4.8 (3.4–6.2)	5.2 (3.0 to 7.5)	0.001	
50-59	25.7 (23.2–28.2)	24.0 (21.5–26.6)	27.5 (24.6–30.5)	27.8 (24.2–31.4)	25.5 (22.1–29.0)	0.2 (-4.1 to 4.4)	0.372	
60-69	71.0 (67.4–74.7)	70.0 (67.4–72.6)	61.9 (58.5–65.3)	66.8 (62.7–70.9)	65.8 (61.0–70.6)	5.2 (-0.8 to 11.2)	0.032	
70-79	99.5 (98.8–100.2)	98.7 (97.9–99.6)	98.7 (97.8–99.7)	97.8 (96.4–99.3)	99.7 (99.4–100.0)	-0.2 (-1 to 0.5)	0.462	
**Sex**								0.651
Male	53.3 (50.8–55.8)	51.1 (47.5–54.7)	52.3 (49.3–55.3)	52.9 (49.8–55.9)	50.3 (46.2–54.3)	3.0 (-1.7 to 7.8)	0.492	
Female	31.2 (28.7–33.7)	30.4 (27.4–33.4)	26.9 (25.4–28.3)	30.0 (27.9–32.0)	28.6 (25.6–31.5)	2.6 (-1.2 to 6.5)	0.198	
**Race/ethnicity**								0.332
N-H White	40.9 (38.8–43.0)	39.3 (36.1–42.5)	37.8 (35.7–39.8)	40.4 (37.8–43.0)	38.0 (34.4–41.5)	2.9 (-1.2 to 7.1)	0.343	
N-H Black	51.0 (47.7–54.4)	50.2 (46.5–53.9)	52.1 (48.8–55.4)	52.5 (48.8–56.1)	53.0 (49.5–56.5)	-2.0 (-6.8 to 2.9)	0.262	
Hispanic	36.4 (31.3–41.4)	36.1 (33.0–39.2)	34.2 (30.3–38.1)	33.2 (30.2–36.2)	31.4 (26.7–36.0)	5.0 (-1.9 to 11.9)	0.072	
Other Race	42.3 (34.9–49.7)	35.9 (27.7–44.0)	34.3 (28.9–39.6)	36.1 (29.7–42.4)	37.8 (32.3–43.4)	4.5 (-4.7 to 13.7)	0.617	
**Education level**								<0.001
< High school	57.5 (53.9–61.1)	56.3 (52.3–60.3)	47.6 (43.8–51.5)	46.7 (42.5–50.8)	45.8 (40.4–51.2)	11.7 (5.2 to 18.2)	<0.001	
High school	47.7 (44.5–50.8)	45.7 (41.3–50.2)	42.3 (38.4–46.3)	45.9 (41.8–49.9)	47.1 (41.8–52.4)	0.5 (-5.7 to 6.7)	0.846	
> High school	33.9 (31.5–36.3)	32.3 (29.2–35.5)	34.6 (32.6–36.7)	37.4 (34.5–40.2)	33.9 (31.3–36.6)	0 (-3.6 to 3.6)	0.15	
**BMI, kg/m^2**								0.29
< 25.0	32.4 (28.9–35.9)	32.2 (29.1–35.4)	31.8 (28.2–35.4)	30.9 (26.9–35.0)	33.4 (28.7–38.1)	-1.0 (-6.9 to 4.8)	0.973	
25.0–29.9	43.7 (41.4–46.0)	42.3 (38.7–45.8)	38.6 (35.8–41.4)	41.1 (37.8–44.5)	38.1 (34.4–41.7)	5.7 (1.3 to 10.0)	0.016	
≥ 30.0	45.6 (42.7–48.5)	43.0 (39.1–46.8)	42.8 (40.0–45.6)	44.9 (42.7–47.1)	41.6 (38.3–44.9)	4.0 (-0.4 to 8.4)	0.314	
**Poverty index** [f]								0.011
< 1.30	53.8 (49.8–57.7)	48.6 (44.8–52.5)	44.2 (41.7–46.7)	48.0 (44.7–51.3)	42.6 (37.3–47.9)	11.2 (4.6 to 17.8)	0.002	
1.30–3.49	49.2 (46.0–52.3)	50.7 (46.7–54.8)	42.0 (37.9–46.0)	45.6 (41.8–49.4)	45.3 (40.8–49.8)	3.9 (-1.6 to 9.3)	0.029	
≥ 3.50	32.3 (29.2–35.3)	30.3 (27.6–33.0)	33.6 (31.1–36.2)	33.7 (30.8–36.5)	32.5 (28.1–36.8)	-0.2 (-5.5 to 5.1)	0.426	
**Insurance status**								0.926
Uninsured	31.2 (26.5–36.0)	32.4 (28.5–36.3)	28.3 (25.0–31.6)	29.7 (24.5–34.9)	31.3 (25.6–37.0)	-0.1 (-7.5 to 7.3)	0.644	
Insured	42.7 (40.7–44.6)	41.0 (38.1–43.9)	40.4 (38.7–42.1)	41.8 (39.5–44.1)	39.4 (36.9–41.9)	3.2 (0.1 to 6.4)	0.168	
**Current smoker**								0.605
No	38.1 (36.0–40.2)	36.2 (33.3–39.1)	36.2 (34.4–37.9)	37.2 (35.3–39.2)	36.5 (33.4–39.6)	1.6 (-2.1 to 5.4)	0.635	
Yes	54.8 (50.9–58.8)	54.6 (51.0–58.3)	50.0 (45.2–54.9)	55.9 (51.5–60.3)	50.3 (45.9–54.7)	4.5 (-1.4 to 10.4)	0.335	

Variable	Weighted Prevalence (95% Confidence Interval), % [b] 2001–2004	2005–2008	2009–2012	2013–2016	2017–2020	Prevalence Change [c], %	P value Linear [d]	Interaction [e]
**Comorbidity**								-
None [g]	12.0 (9.1–14.9)	15.5 (11.4–19.6)	14.8 (11.5–18.1)	14.6 (11.7–17.4)	13.4 (9.4–17.3)	-1.3 (-6.2 to 3.6)	0.704	
Dyslipidemia	48.6 (46.3–50.9)	45.4 (42.9–47.9)	43.3 (41.0–45.7)	46.9 (44.6–49.1)	44.7 (41.8–47.6)	3.9 (0.2 to 7.6)	0.172	
Hypertension	59.8 (57.8–61.8)	57.2 (54.4–60.1)	57.6 (55.2–60.1)	59.4 (56.7–62.1)	58.5 (54.9–62.1)	1.3 (-2.8 to 5.4)	0.979	
Diabetes	77.8 (73.9–81.8)	73.3 (69.4–77.2)	74.1 (70.7–77.6)	76.3 (73.4–79.1)	69.0 (63.9–74.1)	8.9 (2.4 to 15.3)	0.063	

N-H, non-Hispanic; BMI, body mass index.

[a] Elevated cardiovascular risk was defined as a projected 10-year ASCVD risk of 7.5% or greater using the Pooled Cohort Equations.

[b] All estimates were standardized to the 2017–2018 NHANES ASCVD-free adults by the direct method. For males and females, the estimates were age-adjusted, and for other groups, it was age- and sex-adjusted.

[c] Indicates the absolute decrease in the prevalence of elevated cardiovascular risk between 2001–2004 and 2017–2020. Values are percentages (95% CIs).

[d] The statistical significance of a linear trend was assessed using the weighted regression and modeling the midpoint of each time period.

[e] Subgroup differences in trends over time were assessed using the weighted likelihood ratio test by incorporating an interaction term between calendar year and sociodemographic subgroup in the regression models.

[f] Represents the ratio of family income to the federal poverty threshold, adjusting for household size. A higher ratio indicates a higher level of income.

[g] In comorbidity, none was defined as participants without dyslipidemia, hypertension, and diabetes.

Supplementary Table 3. Sex-specific and sex-adjusted prevalence of elevated cardiovascular risk, NHANES 2001–2020 [a]

Variable	Weighted Prevalence (95% Confidence Interval), % [b]					*P* for linear trend [c]	Absolute change [d], %
	**2001–2004**	**2005–2008**	**2009–2012**	**2013–2016**	**2017–2020**		
**Overall**	34.4 (32.8–36.0)	34.2 (31.7–36.7)	34.9 (33.4–36.5)	39.1 (37.0–41.1)	39.5 (37.0–42.0)	< 0.001	5.1 (2.1 to 8.1)
**Sex**							
Male	44.8 (42.1–47.4)	44.4 (40.9–47.9)	48.1 (45.1–51.2)	51.2 (48.1–54.2)	51.2 (47.2–55.2)	< 0.001	6.4 (1.6 to 11.3)
Female	25.3 (23.3–27.4)	25.4 (22.7–28.1)	23.5 (22.1–24.8)	28.5 (26.5–30.5)	29.4 (26.3–32.4)	0.005	4.0 (0.4 to 7.7)
P for group difference	< 0.001	< 0.001	< 0.001	< 0.001	< 0.001		
**Race/ethnicity**							
Non-Hispanic White	33.9 (31.9–35.9)	33.6 (30.4–36.7)	34.2 (32.2–36.2)	39.1 (36.5–41.8)	39.0 (35.4–42.5)	< 0.001	5.1 (1.0 to 9.1)
Non-Hispanic Black	42.9 (39.6–46.2)	44.3 (40.6–48.0)	48.4 (45.0–51.7)	50.9 (47.2–54.5)	53.6 (50.2–57.1)	< 0.001	10.7 (6.0 to 15.5)
Hispanic	29.7 (25.2–34.2)	30.3 (27.3–33.3)	30.4 (26.7–34.1)	31.8 (28.8–34.8)	32.1 (27.4–36.9)	0.347	2.4 (-4.1 to 8.9)
Other Race	35.1 (27.7–42.6)	32.4 (24.5–40.2)	30.8 (25.6–35.9)	34.5 (28.4–40.7)	38.7 (33.2–44.3)	0.251	3.6 (-5.7 to 12.9)
P for group difference	0.002	< 0.001	< 0.001	< 0.001	< 0.001		

NHANES, National Health and Nutrition Examination Survey.

[a] Elevated cardiovascular risk was defined as a projected 10-year ASCVD risk of 7.5% or greater using the Pooled Cohort Equations.

[b] The estimates were sex-adjusted to the 2017–2018 NHANES ASCVD-free adults by the direct method, except for males and females, for whom the estimates were unadjusted.

[c] The statistical significance of a linear trend was assessed using the weighted regression and modeling the midpoint of each time period.

[d] Indicates the absolute increase in the prevalence of elevated cardiovascular risk between 2001–2004 and 2017–2020. Values are percentages (95% CIs).

Supplementary Table 4. Distribution of included and excluded populations, aged 40 to 79 years, NHANES 2001–2020 [a]

	Overall (n = 31,528)	Enrolled [b] (n = 23,594)	With missing data (n = 3,798)	With ASCVD [c] (n = 4,136)
**Sex**				
Male	15,517 (48.0)	11415 (47.4)	1704 (43.3)	2398 (56.7)
Female	16,011 (52.0)	12179 (52.6)	2094 (56.7)	1738 (43.3)
**Age, mean (SD), y**	55.8 (10.6)	54.9 (10.2)	55.2 (10.8)	63.1 (10.0)
**Age group, y**				
40–49	8,932 (33.6)	7415 (36.3)	1137 (36.1)	380 (11.5)
50–59	8,161 (30.9)	6352 (31.8)	1024 (31.6)	785 (23.5)
60–69	8,704 (22.0)	6245 (21.0)	965 (17.9)	1494 (33.5)
70–79	5,731 (13.5)	3582 (11.0)	672 (14.4)	1477 (31.5)
**Year Cycle**				
2001–2004	5,726 (19.6)	4202 (19.2)	713 (22.4)	811 (20.1)
2005–2008	6,248 (21.4)	4772 (21.7)	633 (19.0)	843 (21.1)
2009–2012	6,950 (22.7)	5291 (23.0)	787 (21.1)	872 (21.8)
2013–2016	6,853 (23.9)	5395 (24.7)	641 (18.8)	817 (22.7)
2017–2020	5,751 (12.4)	3934 (11.4)	1024 (18.7)	793 (14.2)
**Race/ethnicity**				
N-H White	13,311 (71.7)	10150 (73.0)	1188 (60.4)	1973 (73.0)
N-H Black	7,371 (10.8)	5156 (9.8)	1198 (16.9)	1017 (12.0)
Hispanic	7,740 (10.9)	6029 (11.0)	879 (12.3)	832 (8.2)
Other Race	3,106 (6.7)	2259 (6.2)	533 (10.4)	314 (6.9)

NHANES, National Health and Nutrition Examination Survey; ASCVD, atherosclerotic cardiovascular disease; N-H, non-Hispanic.

[a] Values are numbers (weighted percentages), unless specified as mean (SD). Interview weight was used in this analysis.

[b] Participants aged 40–79 years with all variables of the Pooled Cohort Equations were enrolled.

[c] ASCVD was defined as participants with any events of coronary heart disease, myocardial infarction, angina, or stroke.

3 Supplementary Figures

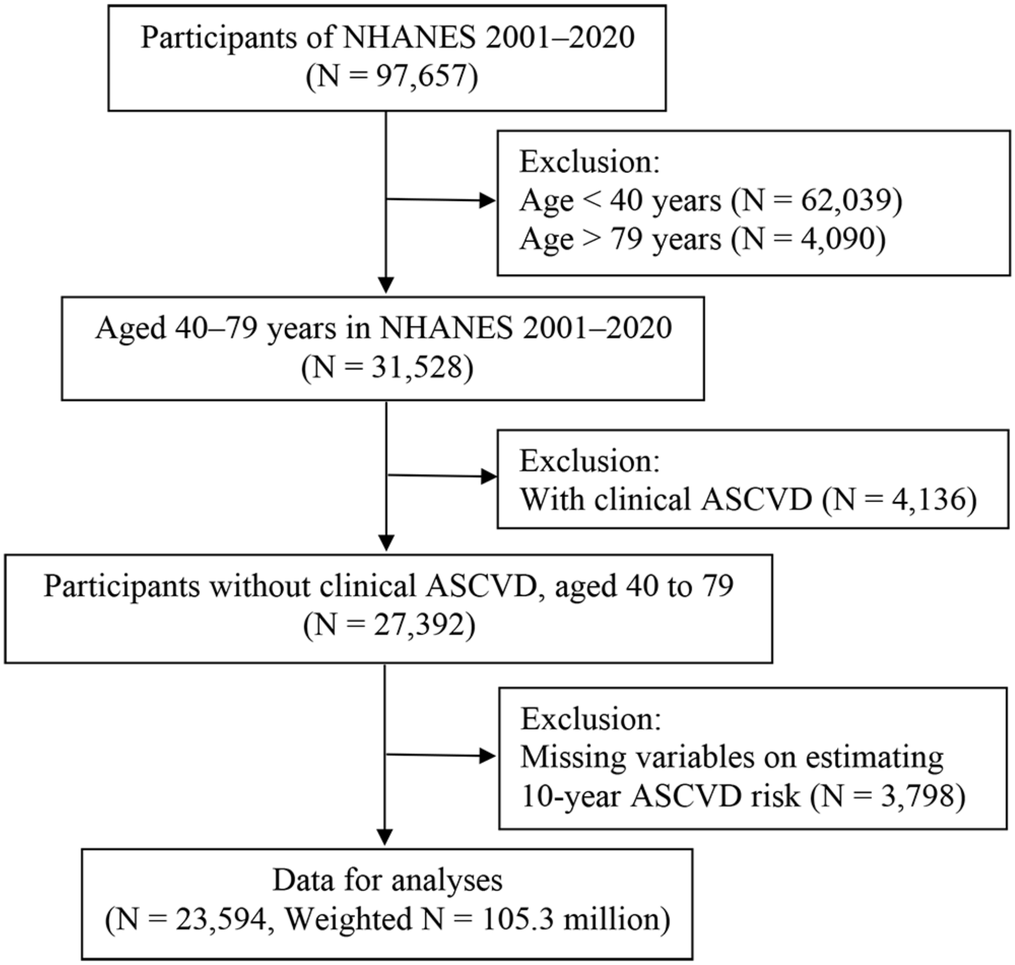

Supplementary Figure 1. Flowchart of participants selection. NHANES, National Health and Nutrition Examination Survey; ASCVD, atherosclerotic cardiovascular disease.

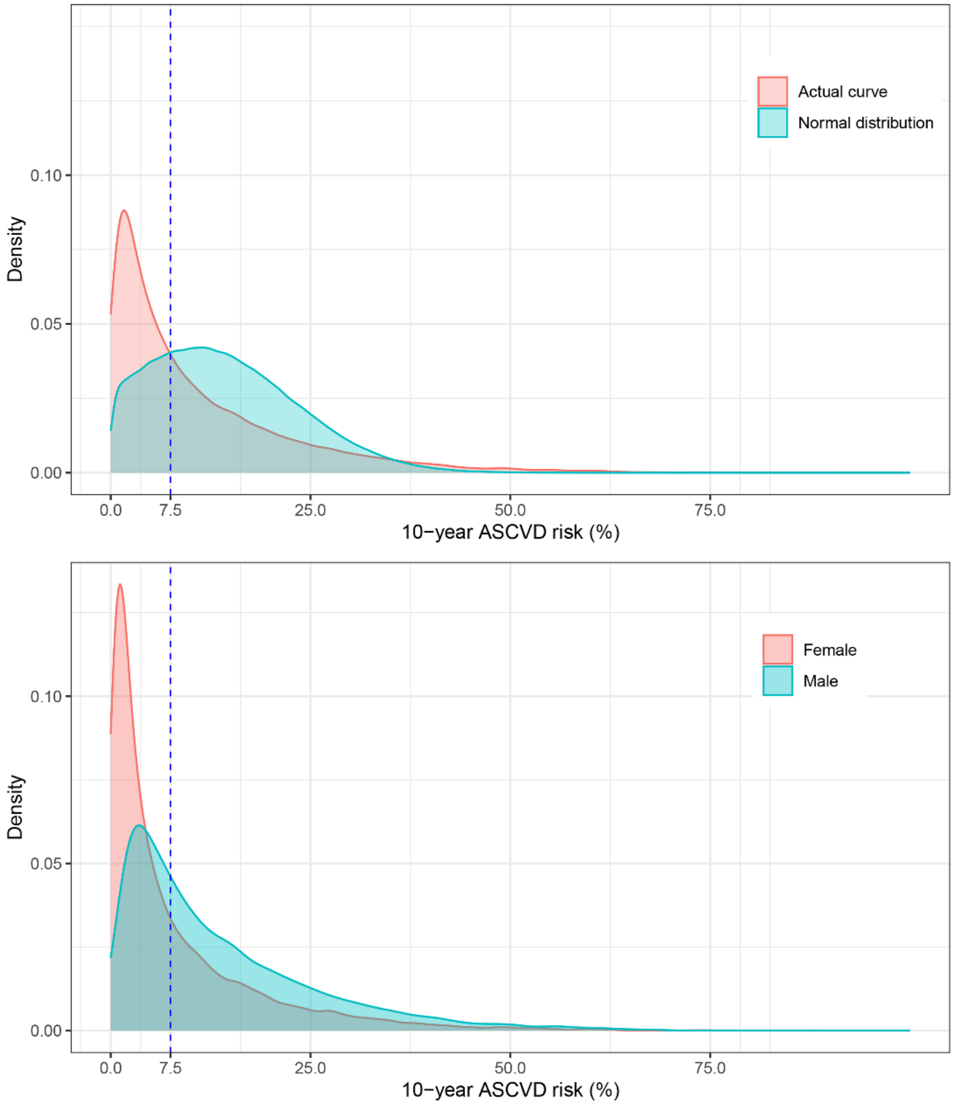

Supplementary Figure 2. Distribution of 10-year ASCVD risk in 2001–2020. ASCVD, atherosclerotic cardiovascular disease. The projected 10-year risk for ASCVD was calculated using the Pooled Cohort Equations. The top panel showed an actual curve of the 10-year ASCVD risk score of all participants from 2001–2020 (pink) and a normal distribution curve using the mean and standard deviation of the 10-year ASCVD risk score of all participants (baby blue). The bottom panel showed the 10-year ASCVD risk score of all participants from 2001–2020 by sex.
